# Repeated Visits to a Woman With Suspected Worms Discharged From the Urethra—A Case Study

**DOI:** 10.1002/ccr3.71071

**Published:** 2025-10-04

**Authors:** Shuqian Cai, Xiaoping Xu, Junchi Xue, Yifei Hu

**Affiliations:** ^1^ Department of Laboratory Medicine Affiliated Jinhua Hospital, Zhejiang University School of Medicine Jinhua China

**Keywords:** artifacts, diagnostic errors, Oligochaeta, urethra, urinalysis

## Abstract

A 58‐year‐old female presented with a 6‐month history of recurrent “worms” found in her toilet, which she suspected originated from her urinary tract. Physical examination was unremarkable. Laboratory analysis revealed motile, blood‐red worm‐like organisms measuring 25–35 mm in length, initially suspected to be *Dioctophyma renale* larvae. However, parasitic screening tests were negative, and microscopic examination identified setae characteristic of oligochaetes. Further investigation revealed these organisms only appeared in the first‐floor toilet. Subsequent sterile urine collection showed no recurrence, confirming environmental earthworm contamination rather than true parasitosis. This case highlights the importance of morphological evaluation and proper specimen collection in differentiating parasitic infections from environmental contaminants.

**Summary**

- When encountering “worms” in urine, consider environmental contamination.
- This case demonstrates how earthworms in toilet water may mimic parasitic infections.
- Microscopic identification of setae and sterile urine collection are crucial for accurate diagnosis, preventing unnecessary treatment.
- Always correlate clinical findings with morphological features to distinguish true parasitosis from artifacts.

## 1 Introduction

The presence of worm‐like organisms in urine often raises concerns for parasitic infections, yet environmental contaminants can mimic true parasitosis, leading to diagnostic dilemmas. *Dioctophyma renale*, a relatively common urinary parasite, shares macroscopic features with common earthworms, potentially causing misdiagnosis. Here, we report a case where repeated detection of motile “worms” in a patient's toilet initially suggested parasitic infection, but meticulous morphological examination and proper specimen collection revealed environmental oligochaete contamination. This case underscores the critical role of microscopic evaluation and standardized sampling in distinguishing artifacts from true pathogens, preventing unnecessary interventions.

## 2 Case History and Examination

**FIGURE 1:**
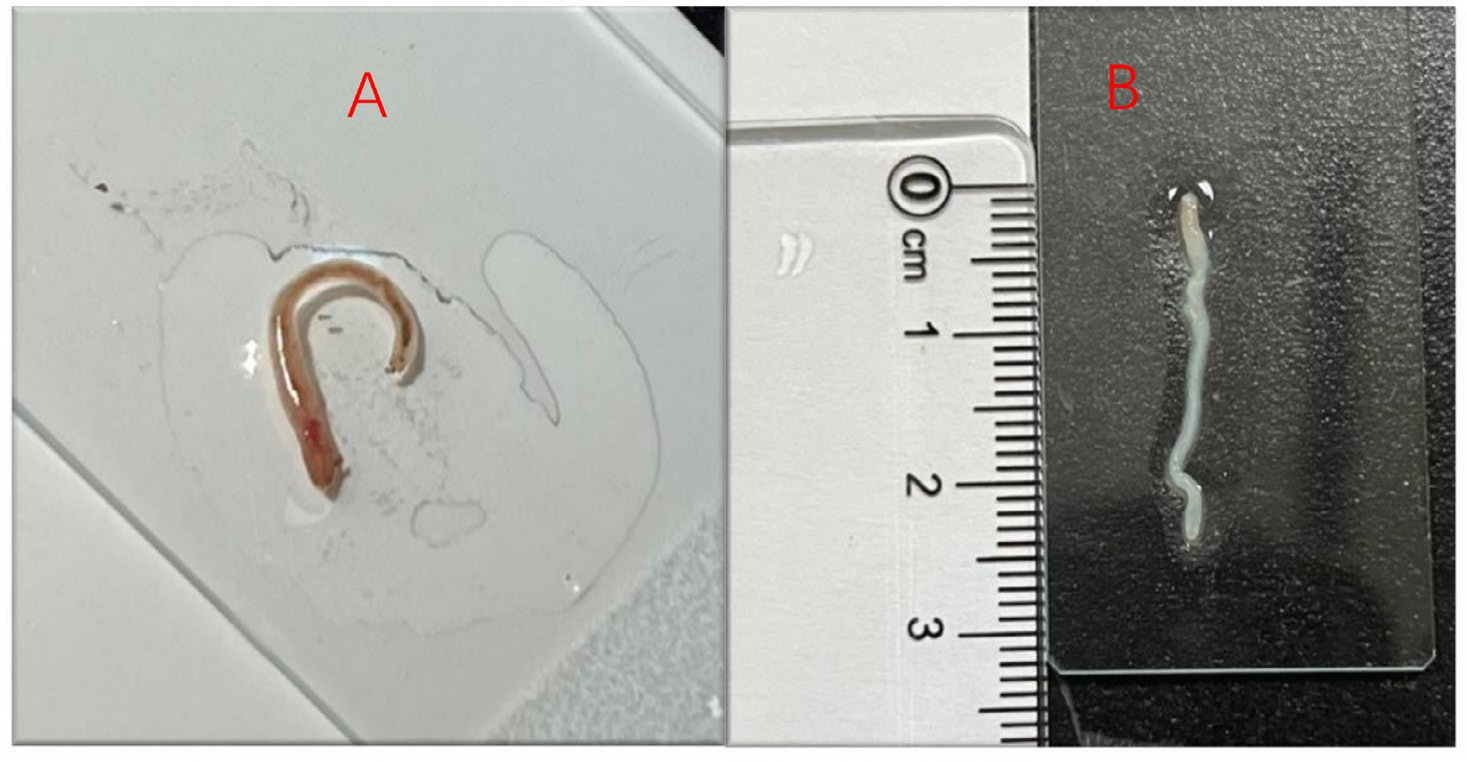
Morphological features of the worm from urine. (A) Live worm (normal saline wet mount) showing blood‐red color. (B) Formalin‐fixed worm with translucent body wall, about 30 mm long.

A 58‐year‐old rural female patient presented to the hospital with recurrent worms in the toilet bowl over a six‐month period and had a recently discovered worm with her at the time of presentation. When asked about her medical history, the patient stated that there had been a total of four instances of worms discharged from the urethra in 6 months, with only one worm discharged each time, and that the worms found on all four occasions were similar in appearance and size. The patient had no hereditary or chronic metabolic diseases and was in good physical condition, with no clinical manifestations such as dull pain in the lower back, frequent urination, urinary urgency, or difficulty in urination. We observed the worms brought by the patient, which were cylindrical in shape, about 25–35 long and 1–2 mm wide, with a blood‐red appearance, and the live worms are shown in Figure 1A. Due to the patient's statement that the worm was discharged from the urethra, and the color and morphology had several similarities with the larvae of the *D. renale*, we initially considered it to be a *D. renale*. Therefore, we performed the following preliminary parasite‐related tests: routine blood test, routine urine test, routine fecal test, total IgE test, urine culture, renal ultrasound, and so on. The routine blood test was within the normal range; the routine urine test was weakly positive, with 3–5 small red blood cells observed in each high‐power microscope field, and no parasite eggs were seen; the routine fecal test for occult blood and parasite eggs was negative; the total IgE value was 1.61 IU/mL (reference range < 190 IU/mL); the test result of schistosomiasis antibody showed negative; the urine culture result was negative; the ultrasound of the kidney was normal.

## 3 Differential Diagnosis and Investigations

Bacteria and fungi are common pathogens associated with urinary tract infections, and urinary tract infections caused by the retrograde passage of adult parasites or eggs are rarely reported. In the literature, there are not many species of parasites excreted in the urine, but the main ones are *D. renale*, pinworms, schistosomiasis, and filariasis, etc. [1, 2], of which *D. renale* accounts for the largest number of reported cases [3]. *D. renale* is blood‐red in color, similar in shape to earthworms and cylindrical in shape; males are (14–35) cm × (0.4–0.6) cm in size, with a bell‐shaped, non‐ribbed symphysis at the end of the tail and a 5–6 mm symphysis spine; females are (20–100) cm × (0.5–1.2) cm in size, with a slightly inflated and bluntly rounded end of the worm, a slightly flattened abdomen, and a slightly elevated dorsal area. The abdomen is slightly flat, and the back is slightly elevated. *D. renale* is mainly found in the kidneys (mostly in the right kidney), and patients may have symptoms such as dull pain in the lower back, renal colic, recurrent haematuria, frequent urination, and urgency of urination; the symptoms are relieved once the nematode is discharged from the body [4, 5]. Although it was initially suspected to be the larvae of *D. renale*, the possibility of infection by *D. renale* was shaken based on the patient's lack of discomfort before and after the expulsion of the worms. The pinworm is milky white in color. The middle part of the worm body is swollen, and the tail end is straight and slender, with a length of approximately 8–13 mm. Its characteristic microscopic structure is the pharyngeal tube ball and wing‐shaped head wing. The worms in this article do not have this structure. The Schistosoma female worm was dark brown. The body was thinner at the front and thicker at the back, with a length of approximately 12–28 mm. No eggs were found in the microscopic examination of urine sediment, and the detection of Schistosoma antibody showed negative results. The filariae are milky white. The microfilariae are slender, about 1.5 mm in length, and need to be observed under a microscope. Based on the above identification points, we basically ruled out the possibility of these parasites.

**FIGURE 2:**
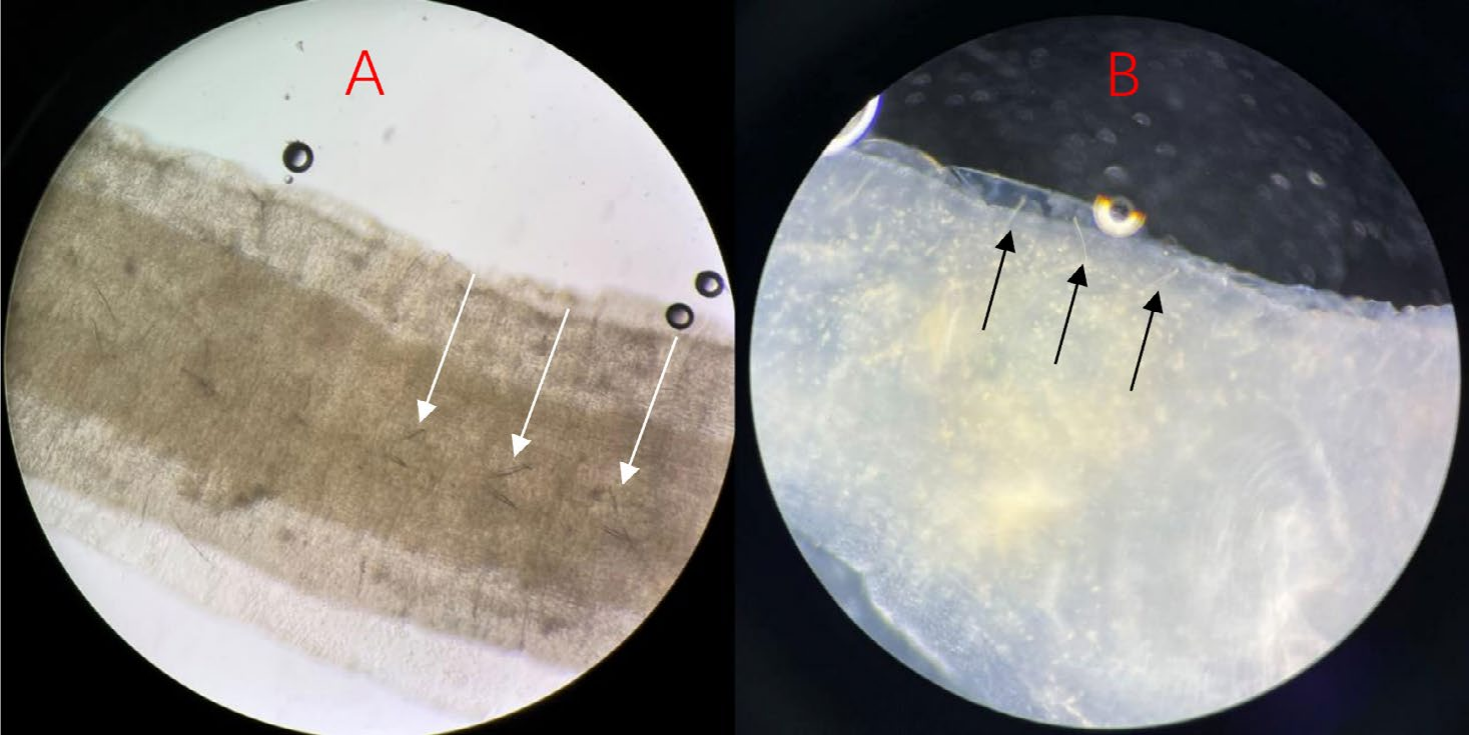
Microscopic setae structures. (A) Ordinary microscopes view (×400) showing aligned setae bundles (white arrows). (B) Phase contrast microscopes view (×400) revealing needle‐shaped setae with basal swelling (black arrow).

In order to identify the source of the worms, we advised the patient to retain a urine specimen in a clean container at home and to continue to send it for examination if the worms were discharged. In the meantime, we fixed the worm in formalin (Figure 1B) and attempted to observe the microstructure of the worm under the microscope to assist in the diagnosis, but unexpectedly found setae structure on the surface of the worm, as shown in Figure 2. Because the setae structure is typical of most oligochaetes and human‐related parasites do not have this particular structure, the hypothesis that the worm was *D. renale* was shaken. To gather further evidence, we consulted the patient's life history: the patient lived in a self‐built house in the countryside, had city tap water for domestic use, disliked eating raw food (such as sashimi), and never ate wild animals, had no pets at home, and lived only with her husband, who had no similar worm infection. All four of the worms were found in a first‐floor toilet, and except for the current worm discovery, which occurred in autumn, the other three worms were discovered in summer.

## 4 Outcome and Discussion

In view of the fact that the patient had no discomfort before or after the exclusion of the worm, no obvious abnormality in the parasite‐related auxiliary examination, no dietary habit of raw food as well as the structure of setae on the surface of the worm under the microscope, we basically ruled out the possibility of *D. renale* and considered that it might be an organism of the natural world. As the body of the worm was made up of links and the surface of the worm had setae structure under the microscope, after reviewing a large amount of literature and the “Atlas of Chinese Animals—Annelids” prepared by Chen Yi et al., the morphology of this worm was consistent with the morphological characteristics of earthworms, and the setae structure on the links was its chitinous bristles. The patient later confirmed that the worm had not been found since the urine was retained in a clean container. On the contrary, the worms were found in the toilet bowl on the ground floor, mainly in the summer, which was consistent with the earthworm's habit of burrowing out of the soil in spring and summer, and it was therefore hypothesized that earthworm larvae in the soil might have entered the toilet bowl from the downpipe, leading to the “farce”. Similarly, Hudson A et al. reported a case in which earthworms were misdiagnosed as pseudo‐parasitic, but in fact originated from an untreated reservoir [6].

Although it was initially suspected to be the larva of the *D. renale*, the possibility of infection by the *D. renale* was ruled out based on the patient's no discomfort as well as the microscopic finding of setae structure on the surface of the worms. After several interviews with the patient, follow‐ups, and a large amount of scientific information, we surmised that the “nematode” came from earthworms in the soil. Earthworms are not human parasites, and their morphological features and identification points are not mentioned in conventional medical textbooks, which undoubtedly poses a great challenge for clinical workers to quickly and accurately identify unknown organisms.

## 5 Conclusion

This case highlights a critical diagnostic consideration in clinical practice: not all worm‐like structures found in urine represent true parasitosis. Through systematic evaluation including proper specimen collection and basic microscopic examination, we identified environmental earthworms mimicking parasitic infection. Simple microscopic examination (e.g., identifying setae) can prevent unnecessary parasitic workup.

## Author Contributions

Shuqian Cai processed the original clinical specimens and wrote the manuscript. Xiaoping Xu consulted literature and participated in revising the manuscript. Junchi Xue contributed to creating figure attachments. Yifei Hu participated in revising the manuscript and provided financial support.

## Ethics Statement

As a single‐case report with the patient's signed consent, no other ethical review was required.

## Consent

Written informed consent was obtained from the patient for the publication of this case report.

## Conflicts of Interest

The authors declare no conflicts of interest.

## Data Availability

The data used in this article are available upon request from the authors.
